# Exploring levers and barriers to accessing primary care for marginalised groups and identifying their priorities for primary care provision: a participatory learning and action research study

**DOI:** 10.1186/s12939-016-0487-5

**Published:** 2016-12-03

**Authors:** Patrick O’Donnell, Edel Tierney, Austin O’Carroll, Diane Nurse, Anne MacFarlane

**Affiliations:** 1 Graduate Entry Medical School, University of Limerick, Limerick, Ireland; 2 North Dublin City General Practice Training Scheme, Catherine McAuley Centre, Nelson Street, Dublin 7, Ireland; 3 National Social Inclusion Office, Primary Care Division, Health Service Executive, Mill Lane, Palmerstown, Dublin 20, Ireland

**Keywords:** Primary healthcare, Marginalised groups, Access, Participatory research, Equity, Patient and public involvement (PPI), Vulnerable groups, Hard to reach

## Abstract

**Background:**

The involvement of patients and the public in healthcare has grown significantly in recent decades and is documented in health policy documents internationally. Many benefits of involving these groups in primary care planning have been reported. However, these benefits are rarely felt by those considered marginalised in society and they are often excluded from participating in the process of planning primary care. It has been recommended to employ suitable approaches, such as co-operative and participatory initiatives, to enable marginalised groups to highlight their priorities for care.

**Methods:**

This Participatory Learning and Action (PLA) research study involved 21 members of various marginalised groups who contributed their views about access to primary care. Using a series of PLA techniques for data generation and co-analysis, we explored barriers and facilitators to primary healthcare access from the perspective of migrants, Irish Travellers, homeless people, drug users, sex workers and people living in deprivation, and identified their priorities for action with regard to primary care provision.

**Results:**

Four overarching themes were identified: the home environment, the effects of the ‘two-tier’ healthcare system on engagement, healthcare encounters, and the complex health needs of many in those groups. The study demonstrates that there are many complicated personal and structural barriers to accessing primary healthcare for marginalised groups. There were shared and differential experiences across the groups. Participants also expressed shared priorities for action in the planning and running of primary care services.

**Conclusions:**

Members of marginalised groups have shared priorities for action to improve their access to primary care. If steps are taken to address these, there is scope to impact on more than one marginalised group and to address the existing health inequities.

**Electronic supplementary material:**

The online version of this article (doi:10.1186/s12939-016-0487-5) contains supplementary material, which is available to authorized users.

## Background

The concept of involving patients and the public in healthcare planning has gained acceptance in recent decades and is enshrined in health policy across a range of international settings [1–7]. The Alma-Ata Declaration of 1978 stated that ‘*people have the right and duty to participate individually and collectively in the planning and implementation of their health care*’, and that effective primary healthcare ‘*requires and promotes maximum community and individual self-reliance and participation in the planning, organization, operation and control of primary health care*’ [8]. This concept of participation continues to capture the attention of health policymakers and planners across both low- and high-income countries today [9–11] and the ‘co-production of health’ and the fostering of ‘equal and reciprocal’ interactions are now seen to be core attributes of health service design [12].

Many benefits of patient and community participation in healthcare planning have been reported, including the improved provision and uptake of initiatives to address health inequalities, the increased acceptance and effectiveness of healthcare services and closer attention to community priorities, and there is also evidence that participatory processes can increase community cohesion and leadership [13–18]. These benefits, however, are not experienced by all, and access to the processes of participation is difficult for many members of society deemed to be ‘marginalised’.

Marginalised groups have been defined as ‘populations outside of “mainstream society”’ [19] and ‘highly vulnerable populations that are systemically excluded from national or international policy making forums’ [20]. Groups commonly described as such include the homeless, drug users, sex workers, refugees, and ethnic minorities such as Roma and Irish Travellers^1^. Many of these groups experience severe health inequities and face significant barriers to accessing high-quality healthcare [21–24]. Consequently, members of these groups often have poorer health status than the general population and inadequate primary care coverage [23, 25–29]. This situation resonates with Tudor Hart’s inverse care law [30] - those most in need of attention by health services are often the least likely to receive that care.

There are many barriers to accessing care for marginalised groups. These include issues relating to the way the health system functions for migrants, homeless people, drug users and people living in poverty [31–36]. Patient factors such as mistrust of services and feeling unwanted have been reported for homeless people, Travellers, drug users and migrants [22, 37–41]. Other barriers seen for particular groups include legal issues for migrants and drug users [22, 42, 43], language barriers for migrants and sex workers [43–46], competing priorities for attention in the lives of homeless people [47], and accommodation issues for those living in deprivation, the homeless, Travellers, drug users and sex workers [38, 41, 48–52]. It is often noted that these barriers do not occur in isolation and that they make patients less likely to reengage with the health services. This aligns with the concept of ‘candidacy’ and the ever-fluctuating relationship between the patient and the health service [53]. Primary care can thus help reduce inequities by acting as a familiar entry point to the wider health system. For this to happen, primary care services that are easy to engage with and acceptable to people from a variety of backgrounds are required [21]. It is rare, however, for these groups to be invited to participate in the planning of primary health services.

Therefore, suitable approaches for engaging with marginalised groups in a constructive way need to be utilised to enable them to highlight their priorities for care. Richard et al. [54] suggested developing co-operative and participatory initiatives to achieve these goals. In this research we sought to do just that - using participatory methods to include the views of a variety marginalised patients on the factors influencing their access to health services. This will then inform the development of more patient centred primary care services that are tailored to their needs.

The overall aim of this participatory study was to involve members of marginalised groups in the development of local primary care services in Ireland by incorporating their views about priority areas for action. This paper reports on the levers and barriers to accessing primary care among a heterogeneous population of marginalised groups, examining a number of shared and differential experiences of accessing primary care and identified priorities for action with regard to that primary care provision.

## Methods

### Study setting

This research was conducted under the auspices of the Partnership for Health Equity (PHE) in Limerick city just as a new Primary Care Team was being established in a socially deprived area of the city (See Table 1).

**Table 1:** Study setting

The Partnership for Health Equity (PHE) is an innovative collaboration which engages medical educators, researchers, clinicians and health service planners from across Ireland in collaboration to work on projects seeking to improve healthcare for marginalised groups. The current partners are the University of Limerick Graduate Entry Medical School, the North Dublin City General Practice Training programme and the Health Service Executive (HSE) Social Inclusion Division. The aim of the partnership is to improve healthcare for marginalised groups by conducting relevant research, by educating future healthcare professionals and by directly providing primary care to marginalised groups. A key feature of the PHE is that research is planned with all partners and research findings are used to inform the development of services, with a focus on priorities for action by the HSE – thereby making real differences in the day-to-day healthcare experiences of patients from marginalised groups across the country.
Limerick City was recognised as the most deprived local authority area in the country in 2014, with 28% unemployment and above average rates for all major causes of mortality (cardiovascular and respiratory disease, cancer, injury) [85]. Groups identified as ‘marginalised’ by the PHE in this setting included migrants, homeless, Irish Travellers, young mothers living in deprived areas, sex workers and drug users.
The Primary Care Team of interest was being established by the HSE and local general practitioners (GPs) in a one of the most deprived areas in the city, with a number of homeless hostels and a high migrant population. The PCT was to consist primarily of a physiotherapist, an occupational therapist, public health nurse (PHN), GPs and allied health professionals.

In keeping with the aims of the national Primary Care Strategy which emphasised community participation in Primary Care Teams [4], and the ethos of the PHE, the health service planners who were PHE members wanted input from marginalised groups on the development of this PCT to identify priorities for action by the team.

The rationale for the work was two-fold. First it was based on our knowledge that marginalised groups are excluded from participatory processes of designing healthcare services despite this being enshrined in health policy (described earlier). Second, there was anecdotal evidence that members of these groups had many barriers precluding them from accessing Irish primary care services despite being entitled to this free government-provided care ‘on paper’ (See Table 2).

**Table 2:** Irish primary care context

To access primary care in Ireland a patient must attend a GP and, if required, be referred to relevant members of the PCT. Patients are required to pay out of pocket to see the GP (cost up to €60 per visit) unless they have a medical card. Applications for this medical card are means tested and the onus is on the patient to find a GP to sign the application form, thereby agreeing to provide care for that patient and to add them to their patient list. This implies that accessing healthcare in the community for low income patients is dependent mainly on a GP accepting a patient’s application. Patients who have been unable to find a GP can apply to the HSE to be assigned to a GP. This medical card covers the cost of visiting the GP and most of the cost of prescription medications. Certain homeless services have access to an ‘emergency medical card’ which allows staff to procure medical care for clients in urgent situations. When a patient with a medical card requires investigations or consultant clinics in public hospitals, there is usually a long waiting time [86]. Patients who pay out of pocket or who have health insurance will often have these appointments arranged much more quickly; this is commonly known as the two-tier health system (for further details see [21]). Government spending on health in Ireland, and other European countries, was reduced during the recession. As O’Donnell et al. [21] have reported in relation to migrant health services, in times of austerity cuts are often made to services targeted at marginalised groups. In 2010 the government in Ireland introduced a ‘prescription charge’ on all medications dispensed from pharmacies to patients with a medical card as a way of saving money in the health service. This levy is currently set at €2.50 per item that the pharmacist must collect on dispensing, i.e. if a patient is prescribed four separate medications for a month they must pay €10 (€2.50 × 4) to the pharmacist. This is an example of an out of pocket payment that seems to disproportionately affect marginalised groups.	

### Research design

This qualitative research was conducted adhering to the interpretive paradigm, and the study design was informed by the principles of Participatory Learning and Action (PLA) research [55, 56]. This methodology came from the work of Chambers [57] in developing country rural settings, and has since been adapted and used urban based primary care projects [55, 56, 58].

PLA is founded on the principles of “democracy, equity, liberation and life-enhancement” [59]. These PLA principles allow groups of participants with varying literacy levels to work together to record and discuss issues relevant to the research question posed [60]. They recognise that participants are experts on their own life experience and they are particularly useful for groups that are typically disenfranchised from involvement in research; for example, migrants and people with aphasia [61, 62]. The participants adopt the role of co-researchers contributing to data generation and analysis. Ethical approval for this study was granted by the Irish College of General Practitioners Research Ethics Committee and the Consolidated Criteria for Reporting Qualitative Research (COREQ) guidelines were followed for reporting of the completed work [63].

### Sampling and recruitment

Following the principles of purposeful sampling [64], the sample was drawn from six marginalised groups: migrants, homeless people, Irish Travellers, drug users, sex workers and young mothers from deprived areas of the city. Participants from these marginalised groups were contacted through gatekeeper organisations known to them or through local HSE PCT members [65]. It is not known how many participants were initially contacted by the gatekeepers, and so it is not possible to identify how many of those contacted agreed to participate in this research. This was in keeping with the ethical approval granted to the research team. The gatekeepers also supported the research team by providing a meeting space familiar to participants for the conduct of the research sessions. All fieldwork took place in community and HSE venues in Limerick city. Shopping vouchers were offered to participants for their time at each research session. Participation in this research was entirely voluntary and a consent form was signed before the first research session. Assistance was given to those with poor literacy at every stage of the consent process.

### Research team

This research was carried out by a multidisciplinary team of three members: two experienced PLA experts (AMacF, a female sociologist and academic who has a PhD; ET, a female research psychologist who has an MA) and one clinician who is experienced in working with marginalised groups (POD, who is a male GP). POD was known to some participants from his clinical work, but he engaged in frequent discussion and reflection on his positionality during this research. It was made clear to participants that POD was at the research sessions in his capacity as a researcher and not as a GP. ET and POD carried out the field work with the support of three GP trainees who took notes for some research sessions. A review of the research question took place with gatekeepers, and piloting of the research question and methods took place with a group of GP trainees prior to starting the formal research. The research team had regular meetings throughout the project to discuss planning issues, engagement with gatekeepers, data analysis and reporting. Updates on the research were provided to the PHE on a regular basis and findings were presented to key stakeholders in the HSE.

### Data co-generation and co-analysis

The fieldwork for this study took place from July 2014 to August 2015. Data were generated by conducting focus groups [66] with four of the six marginalised groups: migrants, homeless people, Travellers, and young mothers living in areas of deprivation. Gatekeepers then advised the research team that individual interviews would be more appropriate for more vulnerable participants from the remaining two groups: sex workers and drug users. This necessitated further discussion with the research ethics committee, and approval for this modification was subsequently granted.

Three PLA techniques were used for the focus groups; these were flexible brainstorming (used in focus group 1), a card sort and direct ranking (both used in focus group 2). A detailed summary of the methods used to facilitate this approach is given elsewhere [62, 67]; Table 3 provides a summary of each technique as it was used in this study.

**Table 3:** PLA techniques

Flexible brainstorming	Fast and creative approach using materials, images and objects to generate information and ideas about accessing primary care
Card sort	An exercise in organising and thematically arranging ideas generated in the flexible brainstorming
Direct ranking	A democratic and transparent process where each stakeholder/participant indicated their priorities or preferences for improving primary care provision

**Fig. 1:**
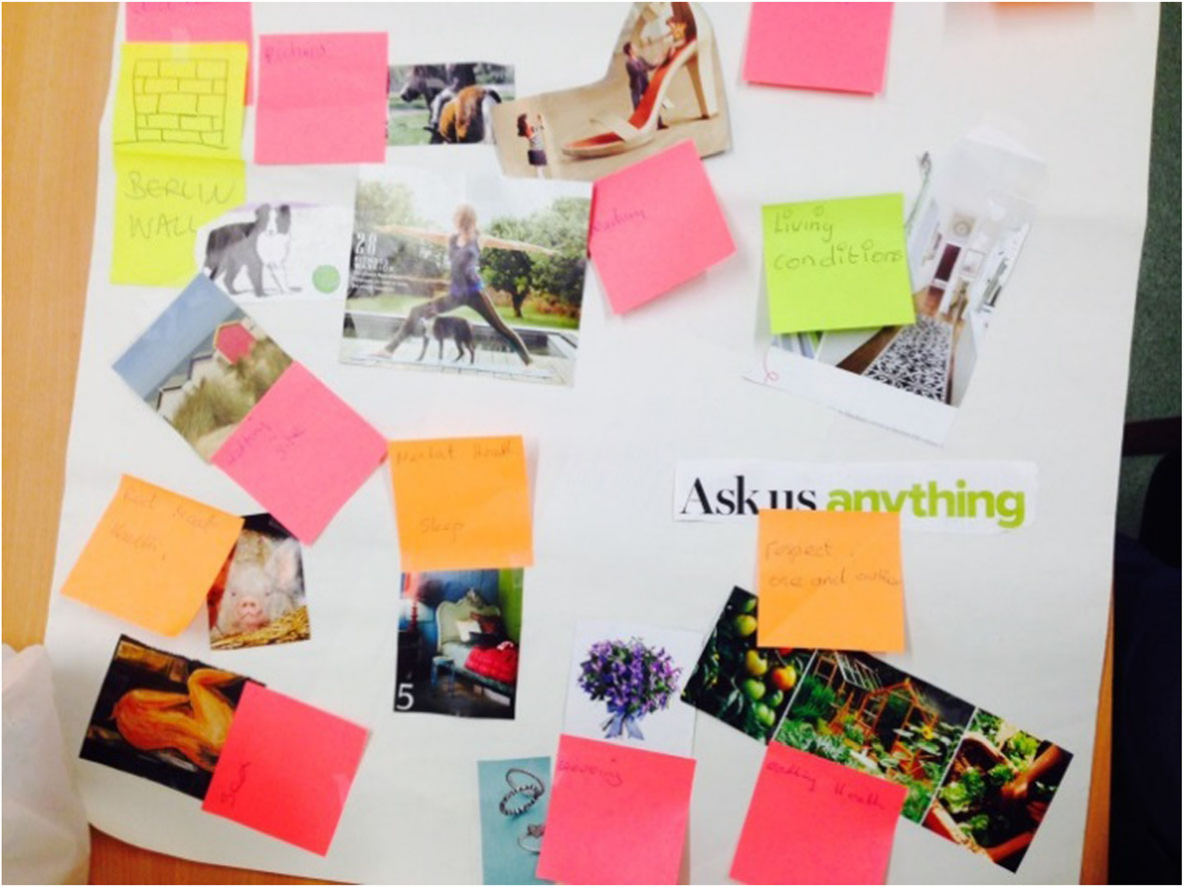
PLA chart after flexible brainstorming

These techniques for generating and analysing data are highly interactive and visual. Participants can record key thoughts on the PLA charts using a variety of materials and these methods are suitable for participants with low literacy levels. Researchers work in collaboration with research participants throughout (see Fig. 1).

The PLA interviews with sex workers and drug users (*n* = 6) used the same methodological approach, but PLA charts were not used. Additional file 1 contains the topic guides for the focus groups and interviews. All research sessions were digitally recorded and field notes and debriefing documents were prepared after each session.

### Thematic analysis

A professional transcription service was employed to produce transcripts of all recordings from the fieldwork. All transcripts and PLA charts were then thematically analysed for overarching themes relating to possible levers and barriers to accessing primary healthcare [68]. Steps for thematic analysis described by Braun and Clarke were broadly followed [69] – including hosting data analysis sessions where all transcripts and PLA charts were displayed allowing immersion in the data, combing the data for themes, then reviewing and refining these themes and using white boards to display their development. Data from the first four marginalised groups were analysed initially and then data from interviews with participants from the two remaining groups were mapped on to these themes to provide a more complete and nuanced description of emergent themes within the data [69]. This reflects the iterative nature of qualitative data analysis: the interview data were used to confirm and validate themes (or not) developed from the focus groups. Taken together this augmented our understanding of the experiences of accessing primary care across a range of marginalised groups. An audit trail of all theme and subtheme arrangements was maintained so that the steps in the analysis were available for scrutiny [70, 71].

### Trustworthiness

An opportunity for all participants to review their contributions as a member checking exercise was offered [72]. For three of the four groups, this took place as a separate meeting where the PLA charts and summary documents were presented to them. None of the interviewees wished to avail of the chance to review their transcripts. There the original PLA charts and a summary document were presented back to the participants. Any modifications or suggestions for changes to the reports were noted by the researchers and consensus was reached by participants on the findings. Recommendations made by the participants at these member checking meetings were incorporated into the data taken for further thematic analysis by the researchers. It is notable that the participants were encouraged to seek ‘shadowed data’ in the form of the opinions of their friends and families on the issues being discussed, and to bring these ideas back to the research sessions [73]. The core research team met regularly to discuss the work, with research notes, transcripts and reflective debriefing notes being circulated and discussed. Designated research data analysis sessions took place to discuss emerging findings across the six marginalised groups.

## Results

A total of 21 participants were recruited across the six marginalised groups involved. Twelve focus group sessions and six interviews were conducted. Participants ranged in age from 19 to 51 years, with an average age of 31 years. Fifteen participants were female and the remaining six were male. Table 4 describes the breakdown of these participants by marginalised group as well as the method of data collection used. The average length of the focus group sessions was 73 min (ranging from 40 to 117 min) and the average length of the semi-structured interviews was 23 min (ranging from 12 to 34 min).

**Table 4:** Overview of marginalised group participants

Participant group	Number of participants	PLA session type	No. of participants who attended more than one PLA session
Migrants	3	3 × focus groups	2
Homeless people	6	3 × focus groups	2
Traveller health advocates	2	3 × focus groups	2
Drug users	3	1 × interview	n/a
Sex workers	3	1 × interview	n/a
Young mothers	4	3 × focus groups	4
Gender: Female	15		
Male	6		
Age average	31 years		
Range	19–51 years		

**Fig. 2:**
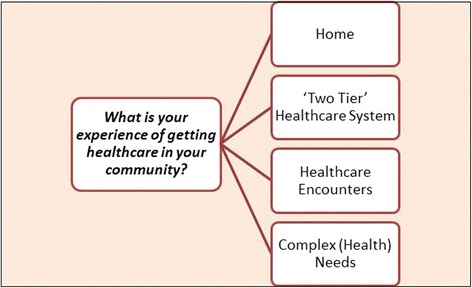
Overview of themes

Four major themes emerged from the focus groups and interviews: the home environment, the two-tier healthcare system, healthcare encounters and complex health needs (see Fig. 2).

These themes are described below, with an emphasis on shared and differential experiences across the groups of access to primary care and their identified priorities for improving primary care provision. Quotes are drawn from two sources: PLA session transcripts (T) or data recorded in written form on PLA charts (C).

### Home

Home was identified as a major theme for three of the marginalised groups included in the research – homeless people, sex workers and Travellers. Data in this theme included references to the accommodation and the general living conditions of the participants, as well as the atmosphere and supports offered to residents of these places including help to access primary healthcare. For some, particularly the homeless and sex worker groups, their accommodation in a homeless hostel often provided them with a positive and nurturing environment.

For the participants living at the homeless hostel, this was primarily a positive experience; they explained that they paid rent which covered meals and single room accommodation, but they also gained access to key worker support and a relatively stable environment. This often allowed the residents to begin reengaging with health services. Hostel staff also worked to find pathways to stable accommodation for the residents.

***‘I found freedom when I came in here anyway. And then you know that they [staff] are trying to help you like, you know it is very positive.’ Homeless participant 1 (T)***

Residents also provided major support to each other, with one resident describing the environment as ‘one big family here’ [Homeless participant 1 (C)]. Many were struggling with addiction and mental illness and they found that peer support with practical issues was invaluable. These issues included making and attending appointments, obtaining medications from pharmacy and even taking prescribed medications regularly.

In stark contrast, the Traveller participants explained feeling that their accommodation on halting sites had a negative effect on the health of inhabitants. They described health problems they felt were due to dampness, poor sanitation and infestations. There was a sense of frustration with the municipal authorities around lack of progress in addressing these issues. Many of the sites were surrounded by high walls; as one participant explained:

***‘You’re locked in. It’s like prison … And if you wanna know, people are suffering seriously with their mental health over it, because depression … no self-confidence, not being able to speak out, because they think they can’t be heard … We had protests and signs up on the wall, [we have] called the Berlin Wall … It’s very wrong.’ Traveller participant 1 (T)***

As a result, Traveller participants described a feeling of being cut off from the local community and the services available, and they questioned whether the authorities were purposely hiding them from the rest of society on these halting sites. They noted that the mental health of residents suffered and stigma was increased due to these living conditions.

In identifying priorities for action, stable accommodation of a certain standard was the key priority for the Traveller participants; they felt that this needed to be addressed before any other health challenges could be looked at. The homeless participants and sex workers also felt the stability they experienced by having reliable accommodation was a priority for them.

### ‘Two-tier’ healthcare system

This theme was concerned with the difficulties created by the structure of the primary care system itself and the confusion around entitlements that can act as a barrier to care. The nature of the ‘opt-in’ system for publicly funded healthcare leaves many marginalised groups at a disadvantage. The obstacles described are perceived to act as a disincentive against engagement by these groups with needs.

Participants across all six groups explained that often the structure of primary care services made it difficult to engage with and access them. One man summarised his thoughts on accessing the public health system by saying: ‘I think [accessing] healthcare should be as easy as making a cup of tea!! But it’s not’ [Homeless participant 4 (C)]. Another participant wrote ‘Access to the information (chaos!)’ [Migrant participant 1 (C)] when describing the difficulty they had finding relevant information on appropriate services and entitlements. Across the groups there was resentment of the level of access and quality of care on offer to patients who could afford private care:

***‘The wealthy get more, [and] get better time from the doctors than people who haven’t got the money for it.’ Homeless participant 1 (T)***

Another participant commented that for easy access to healthcare; ‘it’s all about the money! [you pay]’ [Young mother 2 (C)], and that ‘doctors should treat everybody equally’ [Young mother 2 (C)] whether they were private patients or not.

Difficulty in accessing primary healthcare as a public patient was also discussed by participants; finding a GP to accept them for care was an aspect of the system that posed particular problems for the homeless and drug using groups. Participants perceived this as discrimination. One participant described his attempt to enrol at a GP clinic:

***‘They told me come out [to the clinic], they told me they had space, they told they'd take me on. I went out there, the doctor had a look [at me], said something to the secretary, he went away, the secretary called me, “he's just after letting me know there he's full” [and will not accept you as a patient]. Now I found that with a few [doctors], just not him, that when they saw me … that they didn’t want to know [take me on].’ Drug using participant 1 (T)***

As described in Table 2 of the methods section, to apply for access to the primary care system in Ireland you must complete specific forms and submit documentation. This process was noted to be difficult for many participants across the homeless, drug user and sex worker groups and they often did not complete the process of applying, often leaving them without access to healthcare in the community. One participant explained what this situation meant for her:

***‘I have no medical card … because it went out of date being inside [prison] and I just didn’t get renewed … I need it like; I need to get my teeth fixed and keep an eye on the fucking Hep C, and … when I do come off the stuff [heroin] myself … it won't be too bad after that.’ Sex working participant 1 (T)***

Participants across four of the groups (sex workers, drug users, homeless and young mothers) felt the prescription levy on medication prescribed by the GP was a big problem. One homeless participant called the charge a ‘ransom’ [Homeless participant 1 (C)], and another explained: ‘I really think the €2.50 prescription charge is a real threat to life, I have watched people choose which meds [medications] to take home’ [Homeless participant 4 (C)]. Others revealed that they had not commenced contraception and treatment for infections as they could not pay this charge. Many marginalised patients found this cost forced them to choose between medications and other important expenses:

***‘People … they're getting their dole [weekly welfare payment] … and [out of that money] you're getting your shopping, you're paying your electricity bills and then, and if they're on a lot of medications [this levy is] costing fifteen euro. Like their electricity or their life [medications], do you know what I mean? … they might say oh I'll get my medication during next week and I'll get my electricity now. But they're putting their life in danger then.’ Drug using participant 2 (T)***

Many participants reported that they traditionally relied on community pharmacists for health advice, but for some this relationship has suffered greatly since this prescription charge was introduced as they avoided going to certain pharmacies where they owed money for this levy.

Participants from the young mothers group also mentioned appointment systems in community clinics, particularly in GP clinics, as a barrier to care. They made suggestions for changes that could be made to the eligibility criteria for certain community health services; these factors were identified as priorities for action by this group. Strict referral criteria prevented one participant’s mother from accessing a free chiropodist in the community; she was not old enough to qualify for the service even though she had many chronic illnesses.

The migrant group felt strongly that knowledge concerning the availability of and entitlement to primary care services was a key factor in being healthy and staying well. This was identified as one of their priorities for action in relation to accessing primary care. The rights of patients in relation to making complaints, changing GP, checking qualifications of healthcare providers and requesting information on their own care were noted to be of particular interest:

***‘I think you need to write [information] in black and white; like how do you do it, ABC, because it’s like survival thing.’ Migrant participant 1 (T)***

### Healthcare encounters

This theme includes face-to-face encounters in clinical settings and the experiences of marginalised people in these settings. Past experiences of participants and members of their social networks when engaging with the healthcare system and providers in the community formed an important theme across all groups in the research. Three of the marginalised groups (young mothers, Travellers and migrants) reported having contact with a wide array of healthcare professionals in the community, while others in more vulnerable and disorganised situations often reported less frequent encounters.

Many participants from the groups (migrants, young mothers, drug users and homeless) were adamant that individual healthcare professionals had engaged with them and helped them greatly. One explained about her GP:

***‘She’s worried more about me than I worry about myself!’ Young mother 3 (T)***

Others explained that continuity of care was ideal and seemed to be conducive to attending for care in the community when required. Making access to healthcare professionals as easy as possible was highlighted; the young mothers described a programme where speech and language therapists attended the local crèche to screen for problems. They remarked that this was a big change from the usual system where patients had to try to navigate access to these services by themselves. Others mentioned social workers and pharmacists who had helped them with problems related to their health. Overall, it was the attitudes of the staff that seemed to make people feel welcome and more likely to engage. One participant explained her relationship with a public health nurse:

***‘I think she's marvellous … her attitude is good towards people … even if she's in a rush she'd still look after you … It makes it different because she talks to you, she doesn’t make you feel that you're belittled [not important], you know what I mean? Some people make you feel that they're over you [more important than you]; because they're a doctor or nurse … you should bow down to him. It's not like that down there [PHN clinic], it's like you're the same.’ Drug using participant 2 (T)***

Participants from the migrant group were eager to contrast their engagement with primary care in Ireland to their countries of origin. Some felt that the documenting of full informed consent before any procedure or intervention was very common in the Irish setting. This approach was not always employed for medical interventions in their home country, even for small things such as vaccination, and they found it a positive change that they preferred. Participants also explained that during prior primary care visits they were often subjected to large numbers of tests and investigations for health problems in their home countries, and this contrasted with the ‘relaxed attitude’ [Migrant participant 1 (C)] of the wait-and-see approach employed by many GPs in Ireland.

On the other hand, all participant groups also described negative experiences and encounters with healthcare services and professionals in the community. Poor communication, particularly by GPs, was noted by participants across many of the marginalised groups. This left patients feeling dissatisfied and lacking confidence in the care provided to them. One said of her GP:

***‘He just look into his [computer] system and just prescribe medication for me.’ Migrant participant 2 (T)***

This feeling was echoed by others who noted that GPs often wanted very brief consultations, and even then they were not listening to the health problems being explained to them. This was described as:

***‘A conveyer belt; just in one door and out the other … get your prescription and of out the door with you.’ Homeless participant 2 (T)***

These actions were understood to imply that the doctors were not interested in the problems being presented by these patients. Communication difficulties were magnified for the migrant group, where language problems seemed to increase the frustration felt during consultations. None of these participants noted being offered the services of an interpreter in a primary care setting. Having one present would likely have facilitated better communication during consultations, and avoided this situation:

***‘They [patients] will feel so ignored because they can’t speak English.’ Migrant participant 2 (T)***

However, some of these same participants were sympathetic to healthcare providers and their difficulties with the language barriers:

***‘It’s hard for the patient and GP as well. He doesn’t understand what is the person talking about. So maybe doesn’t understand the problem. So he gives maybe the wrong medicine.’ Migrant participant 3 (T)***

Even with a shared language, the words and phrases used in primary care consultations were noted to be important by the Traveller participants also, as they could easily be misunderstood or misinterpreted. They mentioned that many people had poor understanding of basic health concepts and that often doctors didn’t acknowledge this and take the time to explain illnesses or medications to them. This use of complex medical language could be seen to act as a barrier to accessing care:

***‘When people go to the doctors, [they need] to explain better the big formal words that they uses … [they are] too complicated for people, especially older people … All these fancy [medical] words that they don’t have a clue, unless, if there was one of us sitting with him then fine, but if he [her father] goes in on his own, you might as well be sending a two year old child in [to the doctor].’ Traveller participant 2 (T)***

Unsurprisingly therefore, the need for clear communication tailored to the needs of the patient presenting to primary care was mentioned as a priority for action by the Traveller and migrant groups.

Participants in the homeless and drug using groups who were on opioid replacement therapy (methadone) reported that often when they attended GPs the focus was on the methadone only, and other health concerns were ignored. Also, local pharmacies serve as sites for needle exchange for drug users. One participant recounted that her friend had been asked by a pharmacist what needles she required in front of other customers and this left her feeling:

***‘Ashamed, coming out red faced, looking to see did anyone see you, mortification, do you know what I mean. It's just, it's wrong.’ Drug using participant 2 (T)***

These apparent breaches of confidentiality by staff had stopped her and others from going to certain pharmacies for any health reason. Many of these adverse experiences were described by participants across the groups, and seemed likely to deter them and people in their networks from attending for healthcare appointments in the community. One homeless participant summarised this sentiment well when he said about his GP:

***‘He’s not a man to listen, that’s what I put it down to; that’s why I don’t bother contacting him.’ Homeless participant 2 (T)***

When focusing on priorities and ways to improve these healthcare encounters, many participants across all groups felt that this would be difficult if healthcare providers did not understand the complexity of problems faced on a daily basis by patients from the marginalised groups. One participant said:

***‘It makes me sad that you have to constantly keep explaining yourself and trying to get people to understand your side of it and where you’re coming from, and what’s behind you, and what’s in front of you, and the barriers that’s around.’ Traveller participant 1 (T)***

Without a true understanding of the ‘lived experience’ of these patients, it can be difficult for professionals to improve their access to primary care, and ultimately their health. The view that those working in primary care needed to try extremely hard to understand the difficult lives of these patients was a priority for all participant groups. Another participant explained the huge social distance between the providers and marginalised patients can make engagement and collaboration around health very hard:

***‘Girls that are on the street [sex] working, they'd rather talk to another girl that is working than go and talk to a complete stranger, or a doctor about something that they might have wrong with them … Yeah it's like, you [the doctor] haven't clue what I've been through.’ Sex working participant 2 (T)***

Participants from the migrant group also felt that there needed to be mutual respect for culture in every healthcare interaction and this was suggested as a topic for healthcare professional education; this was therefore a priority for them. Others suggested providers should learn about communication and empathy with marginalised groups:

***‘I think they should talk to … the nurses and doctors and tell them when they're seeing their patients not to be so abrupt with them … when they see their patients; just seeing them as a person and not as a disease … That [patient] person is a person like the doctor, they have feelings, they have to be treated as a human being … For instance, over being on the gear [heroin], I'm frightened to go out to the hospital and down to [the GP to] tell them I'm on the gear; because the attitude [of staff] will change.’ Drug using participant 2 (T)***

### Complex (health) needs

This theme concerns a variety of physical, mental and emotional health issues pertaining to the social determinants of health. Mental health problems were described across almost all of the groups (young mothers, drug users, Travellers and homeless) and many participants spoke of the experiences of their friends and families.

Feelings of stress and anxiety were described by participants in all except the migrant group. Of those with anxiety, many were on prescribed treatments for this, while others explained that they self-medicated with street drugs. This condition impacted on the daily lives of people to such a degree that it made them feel dehumanised; like they were going through the motions of daily life without actually participating in it:

***‘I feel that as well – I feel I exist, I don’t feel I'm living my life.’ Homeless participant 3 (T)***

Depression and self-harm were frequently mentioned, with attempts at suicide being described as a common occurrence:

***‘A lot of people jumped into the river … [it’s now] just everyday kind of thing … It’s like a new craze or something.’ Young mother 3 (T)***

Improving community mental health services for dealing with people in crisis was specifically suggested by members of the young mothers group as a priority for primary care services. One participant mentioned possible actions:

***‘Suicide is a big issue; there should be billboards or more advertising about places [to go for help] … There should be centres for suicide … like for people who are thinking about it, or have thoughts. And they should have more solutions and more funding.’ Young mother 2 (T)***

Some participants from the young mothers group were critical of the ways in which mental health issues, particularly anxiety and depression, were dealt with in primary care. Mental health problems and their management were in fact priorities for the Traveller, homeless, young mothers, drug users and sex worker groups. The young mothers group went further, to recommend certain ways to improve knowledge about community mental health services, and improve their accessibility. They were particularly worried about over-prescribing of anti-depressants and the lack of discussion around alternative treatments for mental health issues, such as relaxation or psychological interventions.

Addiction and the ‘vicious circle’ [Homeless participant 3 (C)] that it can create were documented as being a cause of ill health, but also the associated lifestyle can create barriers to improving health. Living under threat of violence and worries about legal problems were often part and parcel of this existence. The physical effects of drugs and their withdrawal symptoms were described as direct barriers to accessing care in the community. One participant described how her addiction affected her ability to follow medical advice having seen a GP:

***‘The script [prescription] could be still thrown in there [indicating to her bag] two or three weeks later and I wouldn’t have bothered with it … [with] addiction you just, you know what I mean, fuck it, couldn’t give a shit, too busy taking drugs and trying to think of getting money [for drugs], you know?’ Sex working participant 1 (T)***

Discrimination and prejudice experienced by some participants led to feelings of isolation from the rest of society, and this in turn contributed to poor mental health. Anger and despondency were the emotions some participants described when faced with these prejudices. Some participants experienced multiple challenges and forms of marginalisation, as this quote from a participant who was drug using and also sex working shows:

***‘I’m on heroin, and I’ve been struggling with that since I’m fourteen, so I’ve been dealing with things on the street [sex working] then as well, which is really hard, and I got mixed up with that all through my addiction, which I'm not proud of but it's kind of … I mean when you sit at your bed at night like you’re thinking, I’ve all these health issues and you're kind of scared to go [for help] about them. And then when you do go about them, there’s no one that actually wants to listen, that’s the way you feel.’ Sex working participant 2 (T)***

Participants, particularly from the Traveller group, mentioned the importance of tradition and culture in dealing with health issues. Tradition can complicate both the seeking of care in the community and the solutions to health problems that may be suggested. They explained that for their community the concept of privacy and keeping issues within the family were of the utmost importance. It was noted, however, that what was tolerated could change over time with education and discussion. The promotion of tolerance and improved understanding of all groups in society was noted as a way to try to improve this:

***‘We’re all the same, we just come from different ethnic backgrounds … we’re all human at the end of the day; it doesn’t make a difference whether you’re black, white, pink, purple, it – we’re not here to discriminate … we’ve all blood running through our veins, we all have feelings, we all – we just come from different backgrounds and there is serious barriers there, between the guards, the communities, the doctors, the nurses, everybody has their own issues.’ Traveller participant 1 (T)***

Solutions to these complex health problems identified by participants included finding advocates to assist them in accessing healthcare and supporting navigation of the health system. Participants from the Traveller group were themselves on a Traveller Health Advocacy training programme to improve their literacy and advocacy skills along with their basic knowledge of a range of relevant health topics. On completion, they will work to improve the health of their community, and their role is an example of an enabler to accessing primary healthcare:

***‘It involves going out to the Travelling community and meeting people from all different walks of life, it’s about bettering their health, giving them information that maybe they have never received … it’s about [helping] people who can’t read and write and explain to them about what’s on their [information] leaflets … basically what we do is we deliver an awful lot of information out on to the Traveller community.’ Traveller participant 1 (T)***

Participants in the homeless, drug using and sex worker groups spoke about the support of key workers in helping them to try to understand the complexities of the primary care system. Examples of practical supports with making telephone calls, reminders for medical appointments and the provision of transport to appointments were all described in these groups. One said of the supportive relationship she had developed:

***‘I personally deal with a man called B and he's just great, he knows all about the addiction; why would you start on it [heroin] and I mean he meets me and we'd go anywhere for a cup of coffee, sit down. And I notice when you leave [the meeting] then … it's kind of like a breath of relief you know; you say to yourself – that was really nice.’ Sex working participant 2 (T)***

Collaborating with a key worker seemed to remove some of the impediments to accessing primary care discussed previously. Other participants mentioned having transport to clinics, and attending services that offered a comprehensive approach to healthcare for their needs. One example mentioned a location where medical and harm reduction services were co-located:

***‘It’s easy to get to because they [key workers] come and collect you, and bring you to A, and get you back here. Because that’s a big part of stopping you from getting there as well as the, is trying to get there so you know what I mean. It's easier to be picked up and brought … so you have your [addiction] counselling or whatever, the doctor there and your one to ones [needle exchange] all in the one.’ Drug using participant 2 (T)***

The roles of peer advocates and key workers serve as important facilitators to reengagement with the primary healthcare system. It is not surprising then that the homeless group, drug users, sex workers and Traveller participants all mentioned these types of support as priorities.

### Priorities

Some of the priorities for action across the marginalised groups involved in this research have already been highlighted in the description of results. Table 5 provides a synthesis of the priorities identified for each group, and across groups. Some marginalised groups involved in the research recommended specific solutions, and these are also included in the table. It is clear from Table 5 that participants from across marginalised groups had shared views on priority areas for action.

**Table 5:** Priorities for action across the participant groups

Priority Issue	Identified by	Specific solutions suggested to address the priority
Home	• Travellers • Homeless & Sex workers	• Need satisfactory accommodation for any effective primary care engagement to happen • Supports afforded by stable accommodation needed to continue
Two-tier system	• Young mothers • Migrants	• Need for flexibility around eligibility and referral criteria for primary care services • Increased availability of information on entitlements and ways to engage with primary care
Healthcare encounters	• Migrants • Travellers • Migrants & Drug users • All groups	• Better communication in primary care, including availability of trained interpreters • Better communication in primary care; awareness of general literacy and health literacy of patients • Educating professionals on communication skills and empathy • Understanding adversity faced by patients • Show more empathy with the patient
Complex health needs	• Young mothers • Travellers • All groups	• Improved knowledge of and availability of community mental health services • Promotion of tolerance and awareness of prejudice • Supports to access primary care including engaging peer advocates or key workers; modelled on the Traveller group advocacy role

## Discussion

### Statement of principal findings

This study involved 21 members of marginalised groups to investigate their views about access to primary care and to identify priorities for the development of local primary care services. It highlights four key themes in relation to access to primary care for the marginalised groups and priorities for action: the importance of the home environment, the healthcare system and how it is structured, encounters with healthcare professionals, and the complexity of the needs of the groups taking part. Many of the identified barriers were experienced across a number of the marginalised groups and there were similarities in the identified priorities for action across groups. This project demonstrated the effective use of participatory methods for engaging with marginalised groups in a significant way that saw them defining their own needs and priorities.

### Discussion of findings in relation to the literature

The primacy of **Home** and the benefit of having secure and supportive accommodation from the state were articulated by many participants from the homeless and sex worker groups, while for the Traveller group, accommodation they were provided with had a negative effect on the health of inhabitants. This demonstrates that the home environment can act as a springboard to stability and healthcare access and allow a person to begin to address some of the issues they face, or it can contribute to their existing problems. This idea has previously been described in reports and the literature in relation to Irish Travellers, homeless people and drug users [38, 50, 51, 74, 75] and resonates with discussion of the wider social determinants of health [76]. Inter-departmental and inter-sectoral actions to prioritise the creation of stable and supportive home environments for people in the community should be a tenet of any healthcare service.

The **‘Two-Tier’ Healthcare System** that exists in Irish primary care created confusion, which acted as a barrier to care and a source of stress for individuals who were already struggling. The challenges of relying solely on publicly provided care have been reported elsewhere [36]. Further, the finding that the imposition of an ‘out of pocket’ expense, such as the prescription levy, can act as a barrier to medication adherence resonates with the findings of Sinnott et al., who reported on publicly funded patients internationally [77]. We have seen here that this moderate co-payment was seen as a major hurdle for members of marginalised groups. These findings highlight the need for universal healthcare as called for by the World Health Organization [78, 79]. Furthermore, allowing flexibility around referral criteria and appointments for primary care services should be considered when caring for marginalised groups, as suggested by the young mothers group.

Descriptions of **Healthcare Encounters** that are perceived by the patients as poor quality are a recurring theme in the existing literature and were echoed in this study [37, 38]. Communication difficulties were magnified for the migrant group, where language problems increased the frustration felt during engagement with primary care professionals; this has been described previously by Biswas et al. [43] and Newbold et al. [45]. None of the migrant participants in this research spoke about being offered the services of an interpreter in a primary care setting. This echoes findings from O’Donnell et al. [21] and MacFarlane et al. [46] that the provision of interpretation services in Ireland is inadequate. The perceived poor attitudes of primary care professionals towards patients from marginalised groups in this study resonates with findings from many other studies on this topic [32, 33, 37–39, 41, 43, 80–82]. These adverse experiences seem to deter participants in this study from attending for primary care in the community. This finding demonstrates the many complex challenges and layers of marginalisation experienced by these groups and highlights the need for a multifaceted approach to dealing with the issues. Many participants in this study felt that healthcare professionals needed to understand these complexities of the problems facing marginalised groups, and identified professional education in communication skills and empathy as a priority. Without a true understanding of the ‘lived experience’ of these patients, it can be difficult for professionals to improve their access to primary care, and ultimately their health. There is evidence to support Allport’s ‘contact hypothesis’ [83] in relation to these issues; this theory explains that allowing interaction between two very different groups (e.g. homeless patients and trainee GPs) in supervised settings can lead to improved understanding and changed attitudes for both parties.

**Complex (Health) Needs** were experienced by the participants across all groups. Improving community mental health services for dealing with people in crisis was identified as a priority for the young mothers in particular. Accounts of marginalised groups struggling with ‘competing priorities’ in their daily lives have been reported in the literature on homelessness and deprivation [47, 48], but in our work this concept is seen across other groups. The Traveller participants, for example, felt that living in sub-standard accommodation was the primary issue for them to resolve before they could look at enhancing their engagement with primary care services. Others described poor literacy and the subsequent inability to complete applications or understand medication instructions as important problems in relation to their health. These are concrete examples of the social determinants of health in action and highlight that the enhancement of health often requires intensive work on a much broader array of social factors [76]. Being trapped in a cycle of addiction has been reported as a barrier to primary care [41, 75]. This ‘vicious circle’ of addiction compounds the problem of engagement, and primary care professionals need to be aware of these complexities.

Solidarity among members of most of the groups was notable, and reliance on peers who understand the common adversity faced was an important facilitator to navigating primary care access for many. In terms of facilitating healthcare change, some participants described initiatives and agents already working in their communities to try to facilitate effective engagement with primary care. Participants identified advocates who tried to assist them in accessing healthcare and supporting navigation of the health system. Engaging with key workers and peer advocates seemed to reduce some of the obstacles to accessing primary care and facilitate reengagement. Participants from the Traveller group were themselves on a training programme to improve their literacy and advocacy skills along with their basic knowledge of a range of relevant health topics. This model is an example of an enabler to accessing primary healthcare which could be adapted for implementation across other marginalised groups.

While our research focused on interactions with any members of multidisciplinary PCTs, most participants spoke only of meeting GPs, public health nurses or pharmacists. Widening the array of professional support available to marginalised groups can enhance and support health promotion and prevention models of healthcare facilitated by such individuals.

### Strengths and weaknesses of the study

This is one of the first studies to include the voices of a variety of marginalised groups in exploring barriers and facilitators to primary care access with the intention of using these findings to direct action in primary care structures. Various strategies were employed to ensure the rigour of the work, including triangulation, member checking, reflexivity, peer debriefing and reaching data saturation [59, 72]. The collaboration with gatekeeper organisations allowed engagement with some groups traditionally considered ‘hard to reach’ [65]. Participatory methods for working with research participants with varying literacy levels were well received by those taking part. Participants introduced ‘shadowed data’ from their friends and families into the discussions and ideas for the PLA charts; this widened the representation of the marginalised groups [73].

There are also some study limitations. The overall sample size is modest and the sample size for individual marginalized groups is small. The sample is also from one urban setting only. This does limit the range of experience being offered for analysis and used to identify priorities and, as with all qualitative studies, raises questions about the credibility and transferability of findings. However, the overall sample of 21 and the repeated engagement of several participants are positive given the complex and chaotic lives being lived. Also, the resonance of key findings with previous literature (described earlier) suggests that the findings have authenticity and relevance for service planning.

The PLA methods used with the focus groups ideally require repeated meetings over a period of time, thereby allowing participants to reflect and discuss the research with others in their networks. Arranging the schedules of research was difficult in some cases due to the chaotic lives of some of the participants; crises including the sudden death of one participant between meetings meant adaptations had to be made. Despite maximal flexibility of the research team and the gatekeepers it was not possible to have the same participants present at all research sessions. Finally, all participants in this research were clients of gatekeeper organisations and so had some history of contact and engagement in an effort to improve their lives. They may therefore be seen as having more knowledge and resources to access primary care than other members of the same marginalised groups who are not working with gatekeepers.

### Clinical and policy implications

The findings from this study have a number of clinical implications. Ineffective styles of communication used by healthcare practitioners were highlighted as a barrier to healthcare access, and so the education of these front-line professionals and their support staff on relevant skills should be prioritised. Making appropriately qualified interpreters available to patients who require support with language in all primary care settings is another recommendation on the issue of communication.

Widening the access for marginalised groups to other members of the PCT beyond GPs and PHNs should be considered, as well as flexibility around referral and eligibility criteria for accessing certain primary care services. Key worker organisations and advocacy programmes should be resourced to allow them to work to facilitate the navigation of primary care by members of marginalised groups.

Government levies such as the ‘prescription charge’ seem to disproportionately affect the marginalised groups we researched, and exemptions should be considered for these groups. The HSE Social Inclusion Division was instrumental in recently securing an exemption for asylum seekers from paying prescription charges. Simplifying the system of application for and retention of medical cards would also help many vulnerable patients to engage with primary care [84]. Removing the link between being granted a medical card and finding a GP to accept you as a patient would allow many members of marginalised groups a certain degree of access to primary care. Lastly, attempting to address basic needs such as education and housing is important for all of society, but particularly in relation to the health of marginalised groups.

### Areas for further research

It would be valuable to analyse marginalised groups’ experiences of participatory learning and action research methods and to explore how they experience them compared with other research studies that they may have been involved in. It would also be interesting to use PLA to work with marginalised groups to explore their experiences and priorities in relation to secondary care and access to aspects of social care. The cost effectiveness of designing interventions that address a priority issue for a number of marginalised groups should be evaluated. For example, healthcare professional education on communication skills and competencies for working with a wide variety of marginalised groups could be developed and evaluated from this perspective. Innovative ways of improving access to primary care services for marginalised groups, such as peer support networks, should be explored and evaluated.

## Conclusions

There are many complicated personal and structural barriers to healthcare access shared across a number of marginalised groups. They also have shared views on priority areas for action. If steps are taken to address these priorities, there is scope to impact on more than one marginalised group and to address the existing health inequities.

## Additional file

Additional file 1: Research Topic Guide. (DOCX 15 kb)

## Abbreviations

COREQ: Consolidated Criteria for Reporting Qualitative Research
GP: General practitioner
HSE: Health Service Executive
PCT: Primary Care Team
PHE: Partnership for Health Equity
PHN: Public health nurse
PLA: Participatory Learning and Action
PPI: Patient and public involvement

## Footnotes

1 Irish Travellers are defined as ‘the community of people who are commonly called Travellers and who are identified (both by themselves and others) as people with a shared history, culture and traditions, including historically, a nomadic way of life on the island of Ireland’ [87].
