# Supplementary material for: Exploring levers and barriers to accessing primary care for marginalised groups and identifying their priorities for primary care provision: a participatory learning and action research study
Source: Int J Equity Health. 2016 Dec 3;15:197. doi: 10.1186/s12939-016-0487-5 (PMC5135741; doi:10.1186/s12939-016-0487-5)
Supplement: Additional file 1: — Research Topic Guide. (DOCX 15 kb) [file 12939_2016_487_MOESM1_ESM.docx]

## Research Topic Guide

What is your experience of getting health care in your community?

Think about getting health care from ….GP, Public Health Nurse, Chiropodist, Physiotherapist etc.

How easy or difficult is it to go to see them?

What makes it easy or hard?

Tell me about the last time you visited your GP or other health care professional?

Think about other people you know: your family, your network, other e.g. friends etc.; what are their experiences?

What is the most important thing that you would like to us to tell the HSE who plan community health services?

- What is most important thing or issue for the implementation group (IG) to hear?
- What is the least important thing for the implementation group to hear
- Remember these are the people who may be able to do something to change the situation
